# IL-27 mediates HLA class I up-regulation, which can be inhibited by the IL-6 pathway, in HLA-deficient Small Cell Lung Cancer cells

**DOI:** 10.1186/s13046-017-0608-z

**Published:** 2017-10-11

**Authors:** Grazia Carbotti, Amin Reza Nikpoor, Paola Vacca, Rosaria Gangemi, Chiara Giordano, Francesco Campelli, Silvano Ferrini, Marina Fabbi

**Affiliations:** 1 Ospedale Policlinico San Martino, IRCCS for Oncology, 16132 Genoa, Italy; 2 Immunogenetic and Cell Culture Department, Immunology Research Center, School of Medicine, Mashhad University of Medical Sciences, Mashhad, 919677-3117 Iran; 3 Immunology Research Area, Bambino Gesù Children’s Hospital, IRCCS, Rome, Italy; 4 Ospedale Policlinico San Martino, UOC Bioterapie, Largo R. Benzi 10, 16132 Genoa, Italy

**Keywords:** IL-27, IL-6, Small Cell Lung Cancer, HLA class I, PD-L1, STAT1/3, SOCS3

## Abstract

**Background:**

Recently, immunotherapy with anti-PD-1 antibodies has shown clinical benefit in recurrent Small Cell Lung Cancer (SCLC). Since anti-PD-1 re-activates anti-tumor Cytotoxic T Lymphocyte (CTL) responses, it is crucial to understand the mechanisms regulating HLA class I, and PD-L1 expression in HLA-negative SCLC. Here we addressed the role of IL-27, a cytokine related to both IL-6 and IL-12 families.

**Methods:**

The human SCLC cell lines NCI-N592, -H69, -H146, -H446 and -H82 were treated in vitro with different cytokines (IL-27, IFN-γ, IL-6 or a soluble IL-6R/IL-6 chimera [sIL-6R/IL-6]) at different time points and analyzed for tyrosine-phosphorylated STAT proteins by Western blot, for surface molecule expression by immunofluorescence and FACS analyses or for specific mRNA expression by QRT-PCR. Relative quantification of mRNAs was calculated by the ΔΔCT method. The Student’s T test was used for the statistical analysis of experimental replicates.

**Results:**

IL-27 triggered STAT1/3 phosphorylation and up-regulated the expression of surface HLA class I antigen and of *TAP1* and *TAP2* mRNA in four out of five SCLC cell lines tested. The IL-27-resistant NCI-H146 cells showed up-regulation of HLA class I by IFN-γ. IFN-γ also induced expression of PD-L1 in SCLC cells, while IL-27 was less potent in this respect. IL-27 failed to activate STAT1/3 phosphorylation in NCI-H146 cells, which display a low expression of the IL-27RA and GP130 receptor chains. As GP130 is shared in IL-27R and IL-6R complexes, we assessed its functionality in response to sIL-6R/IL-6. sIL-6R/IL-6 failed to trigger STAT1/3 signaling in NCI-H146 cells, suggesting low GP130 expression or uncoupling from signal transduction. Although both sIL-6R/IL-6 and IL-27 triggered STAT1/3 phosphorylation, sIL-6R/IL-6 failed to up-regulate HLA class I expression, in relationship to the weak activation of STAT1. Finally sIL-6R/IL-6 limited IL-27-effects, particularly in NCI-H69 cells, in a SOCS3-independent manner, but did not modify IFN-γ induced HLA class I up-regulation.

**Conclusions:**

In conclusion, IL-27 is a potentially interesting cytokine for restoring HLA class I expression for SCLC combined immunotherapy purposes. However, the concomitant activation of the IL-6 pathway may limit the IL-27 effect on HLA class I induction but did not significantly alter the responsiveness to IFN-γ.

**Electronic supplementary material:**

The online version of this article (10.1186/s13046-017-0608-z) contains supplementary material, which is available to authorized users.

## Background

Small Cell Lung Cancer (SCLC) is an aggressive tumor characterized by rapid and extensive metastatic dissemination, recurrence after chemotherapy and poor prognosis. Therefore, there is an urgent need of new treatment modalities, and promising results have been achieved in recent phase I-II studies of immunotherapy [1]. In general, immune checkpoint blockade through monoclonal antibodies targeting PD-1, PD-L1, and/or CTLA-4 have shown unprecedented activity in several metastatic malignancies, including melanoma and Non-Small Cell Lung Cancer (NSCLC) [2–4]. A recent phase I-II trial of the anti-PD-1 antibody nivolumab in patients with recurrent SCLC showed a 10% response rate and a 32% disease control rate [5]. In addition, different schedules of nivolumab in combination with ipilimumab showed 19–23% response rates [5]. These results prompted the National Comprehensive Cancer Network to consider the nivolumab-ipilimumab combination in the 2016 guidelines for SCLC treatment.

It is now well established that immune checkpoint blockers re-activate pre-existing, silenced CTL cell responses against tumor neo-antigens, in metastatic melanoma and NSCLC [6–8]. However, SCLC is a tumor lacking HLA class I expression and, accordingly, should be resistant to the activity of HLA-restricted CTLs [9]. Nonetheless, IFN-γ is capable to restore HLA class I expression [10] and sensitivity to CTL-mediated recognition of SCLC [11, 12], as well as of other tumor cells showing down-regulated HLA class I antigen expression [13]. In view of its immune-modulatory and direct anti-tumor effects, clinical studies of IFN-γ have been performed in different cancers, with some evidence of activity in ovarian and bladder cancer. However, no activity was found in other cancers and adverse effects including toxicity or even tumor progression have been recorded (reviewed in [14]). These findings may relate, at least in part, to the ability of IFN-γ to activate immune-regulatory loops, for example through the induction of PD-L1 or indoleamine 2,3 dioxigenase (IDO) [15].

We recently reported that IL-27, a heterodimer cytokine related to both IL-6 and IL-12 cytokine families [16, 17], has several functional activities in common with IFN-γ, in different cancer cells [18]. Indeed, IL-27 up-regulates multiple components of the HLA class I antigen presentation machinery in human cancer cells, thus facilitating Cytotoxic T Lymphocyte (CTL) recognition. Moreover, in lymphoid cells, IL-27 induces the expression of the transcription factor T-bet, an inducer of Th1 and CTL responses [19], which have been involved in the anti-tumor activity of IL-27. IL-27 may also exert direct anti-tumor effects through the inhibition of angiogenesis and neoplastic cell proliferation in different cancers including acute myeloid leukemia [20], prostate cancer [21], and melanoma [22]. In addition, IL-27 inhibits the expression of stem cell and mesenchymal transition genes in NSCLC cells [23, 24]. In view of its immune-enhancing activities and direct anti-tumor effects IL-27 has been considered as a potential anti-tumor agent [25]. On the other hand, IL-27 induces the expression of immune-regulatory molecules such as the IL-18 natural inhibitor, IL-18BP [26], the tryptophan catabolic enzyme IDO and PD-L1 [27], in neoplastic cells. Therefore, IL-27 may have a dual role in anti-tumor immunity [28] and shares several immune-regulatory functions with IFN-γ, in relationship to the common usage of the STAT1 intracellular signaling pathway [18, 19, 23, 29].

At the best of our knowledge, no studies have addressed the effects of IL-27 on SCLC cells, so far. In this study we tested the effects of IL-27 on a panel of SCLC cell lines and found that it is capable to restore HLA class I expression through the up-regulation of peptide transporters and other components of the class I antigen presentation machinery in most SCLC lines tested. We also explored the expression and signaling properties of the IL-27 receptor complex, a heterodimer of IL27RA/WSX1 and GP130 chains [30]. IL-27 and IL-6 share the usage of the GP130 chain and downstream signaling pathways through STAT1/3 [30]. In addition, serum IL-6 is elevated in SCLC in relationship with advanced stages, worse prognosis and Neuron Specific Enolase (NSE) levels, suggesting a possible role of IL-6 in SCLC progression [31], similarly to other cancers [32]. Therefore, we also tested the possible effects of IL-6 on SCLC cells and dissected the role of the GP130 molecule by the usage of a sIL-6R/IL-6 synthetic ligand, similar to “hyper-IL-6” [33], in both IL-27-responsive and unresponsive SCLC cells.

## Methods

### Cells and treatments

The human SCLC cell lines NCI-H69, NCI-H146, NCI-H446, NCI-H82 were purchased from ATCC and NCI-N592 was kindly provided by Dr. J. Minna (NCI, Washington DC). Cells were grown in RPMI 1640, with L-glutamine, 10% FCS and antibiotics (Lonza) and never kept in culture for longer than 4 months, when an aliquot of the original stock was thawed. Treatments with cytokines were performed with slight differences, according to the final use of the stimulated samples. Conditions were set on the bases of preliminary titration experiments. For immunofluorescence and QRT-PCR analyses, cells were seeded in 24-well plates in culture medium at 5 × 10^4^ cells/well and different cytokines were added: IFN-γ (1000 IU/ml, PeproTech, 300–02), IL-27 (100 ng/ml R&D System, 2526-IL-010), IL-6 (50 ng/ml R&D System 206-IL-010) or recombinant human IL-6Rα/IL-6 chimera [sIL-6R/IL-6] (50 ng/ml R&D System 8954-SR-025). Treatments were carried out for 48 h.

For the analysis of tyrosine-phosphorylated STAT proteins, 1 × 10^5^ SCLC cells were incubated in a test tube at 37 °C with or without 50 ng/ ml of IL-27, 20 ng/ml of IL-6, 40 ng/ml of sIL-6R/IL-6 in 0.5 ml of medium for the 10, 30 or 60 min time points. Incubations for the 1, 2, 3 or 4 h time points were carried out in 1 ml culture medium. Treatments for the 6, 18 and 24 h time points were performed in 24-well plates, in 1 ml culture medium. Cells were then rescued by centrifugation and immediately processed.

### Immunofluorescence

Immunofluorescence with anti-GP130 PE, anti-IL-27RA/WSX1/TCCR APC (R&D Systems, Clones 28,126 and 191,116), anti-PD-L1 PE or Isotype Control PE (eBioscience Bender, BMS-125983-41 and BMS-124724-41, respectively), anti-human IL-6Rα FITC (R&D Systems, Clone 17,506 FAB227F-025) was performed according to manufacturer’s instructions. Indirect immunofluorescence was performed on 5 to 10 × 10^4^ cells/sample with anti-HLA class I W6/32 mAb (ATCC) and FITC-labeled goat anti-mouse (Jackson Immunoresearch, 115–096-068) according to standard techniques. Cell fluorescence was analyzed by flow cytometry with a FACScan (Becton & Dickinson) using the Cell Quest software or a Gallios (Beckman Coulter). Gating on viable cells was performed using physical parameters and 10^4^ gated events were acquired.

### Western blot

Cells were lysed in lysis buffer (20 mM Tris-HCl pH 7.4, 1 mM EDTA, 150 mM NaCl, 1% Brij97) containing 2 mM Na Orthovanadate and protease inhibitors (Roche Diagnostics, Complete Mini 04693124001). Lysates were resolved under reducing conditions by SDS-PAGE (10% or 13% acrylamide) and analyzed by Western blotting using the following antibodies: rabbit anti-phospho-STAT1 (pY701) and anti-STAT1 anti-sera (Cell Signaling Technology, 9167 and 9172, respectively), murine anti-phospho-STAT3 (pY705) and anti-STAT3 mAbs (BD Transduction Laboratories, 612,356 and 610,190, respectively), rabbit anti-SOCS3 (Cell Signaling Technology 2932) and murine α-tubulin or β-actin mAbs (Sigma-Aldrich T6074 and A2228, respectively). Proteins were detected by ECL Prime (GE Healthcare, RPN2232) and visualized by a chemiluminescence gel documentation and analysis system (MINI HD, UVITEC, Cambridge).

### RT-PCR analysis

Total RNA was isolated by the NucleoSpin RNA kit (Macherey-Nagel, 740,955.250) and reverse-transcribed using the SuperScript III Reverse Transcriptase (Invitrogen, 18,064–071). Amplification was carried out by the Mastercycler® ep realplex4 instrument (Eppendorf International) using the iQTM SYBR® Green Supermix system (Bio-Rad Laboratories, 170–8882). Quantification of mRNAs relative to housekeeping gene was expressed as 1/ΔCT. Expression levels of mRNAs relative to untreated control were calculated by the ΔΔCT method.

### Statistical analysis

Data are expressed as the mean ± standard deviation (SD) of triplicates. The Student’s T test was used for the statistical analysis of experimental replicates. A value of *p* < 0.05 was considered significant.

## Results

### IL-27 up-regulates surface HLA class I expression in SCLC cells

**Fig. 1:**
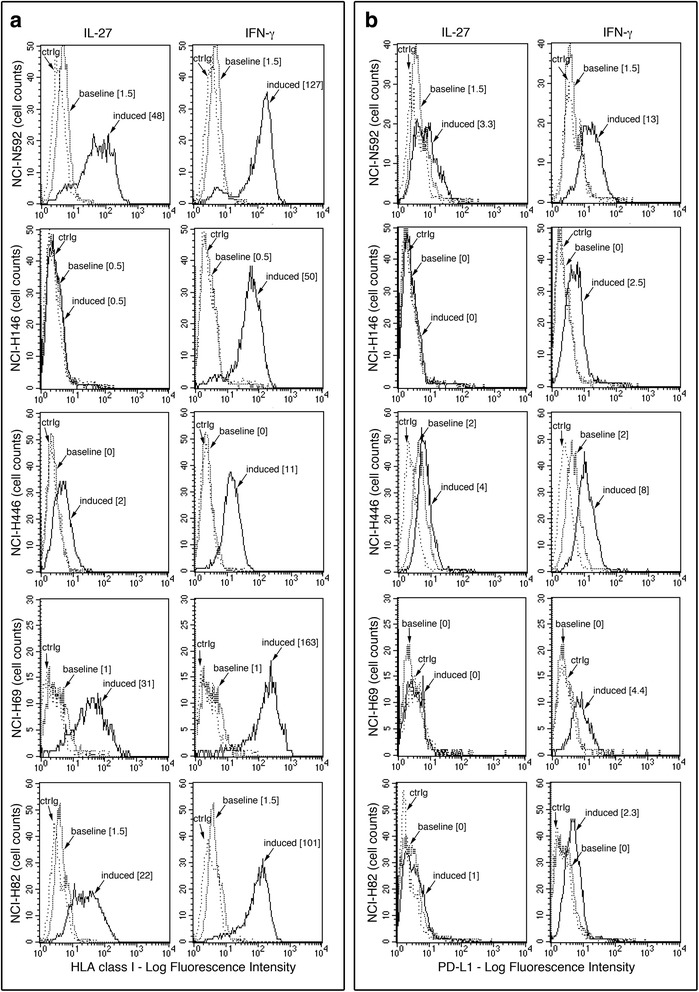
Effect of IL-27 on surface HLA class I and PD-L1 expression in human SCLC cells. Cytofluorimetric analysis of membrane HLA class I (**a**) or PD-L1 (**b**) expression in five SCLC cell lines, cultured in the presence of medium (baseline), IL-27 or IFN-γ (induced), for 48 h. Isotype-matched Ig control is indicated (ctrIg). Numbers in brackets represent Median Fluorescence Intensity (MFI) values calculated as median anti-HLA class I (W6/32), or anti-PD-L1 mAb minus median Ig control

In view of the defective expression of HLA class I molecules in SCLC [9], we tested whether IL-27 could up-regulate membrane HLA, as recently reported in other tumor cell types [18]. To this end, we cultured a panel of 5 SCLC cell lines for 48 h with IL-27 or IFN-γ, a known inducer of HLA expression, as control. SCLC cells were then analyzed by indirect immunofluorescence and flow cytometry using the W6/32 mAb, which recognizes HLA class I heavy chains in complex with the β2-microglobulin. Four out of five IL-27-stimulated cell lines showed up-regulation of HLA class I molecule expression, while NCI-H146 cells appeared resistant (Fig. 1a). All SCLC cell lines including the IL-27-resistant NCI-H146 cell line efficiently responded to IFN-γ by up-regulating their surface HLA class I expression, in agreement with previous reports [10, 11]. In general, the effect of IFN-γ on HLA class I expression was stronger than that of IL-27, even on IL-27-responsive cell lines.

We tested also surface PD-L1 molecule, which is inducible by both IL-27 [27] and IFN-γ [15], in different tumor cell types. As shown in Fig. 1b, IL-27 only weakly induced PD-L1 expression in NCI-N592 and NCI-H446 cells, whereas IFN-γ could increase PD-L1 expression in all the five cell lines tested. Notably, IFN-γ up-regulated PD-L1 expression also in the NCI-H146 cells, which are resistant to IL-27 effects.

**Fig. 2:**
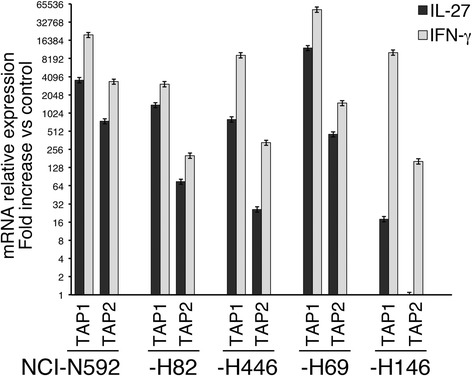
IL-27 increases mRNA expression of *TAP1* and *TAP2* genes. QRT-PCR analysis of *TAP1* and *TAP2* mRNA expression in IL-27- and IFN-γ-stimulated cells relative to untreated controls from five SCLC cell lines (NCI-N592, -H82, -H446, -H69 and -H146). Cells were cultured in the presence of medium, IL-27 (black histograms) or IFN-γ (grey histograms) for 18 h. Data, normalized to *GAPDH* housekeeping gene, are expressed as fold change relative to control. Error bars represent SD in one representative experiment out of two with consistent data

The effect of IFN-γ on HLA class I expression has been related predominantly to the up-regulation of *TAP1* and *TAP2* gene expression [11]. Here, we show that also IL-27 clearly up-regulated both *TAP1* and *TAP2* mRNA expression in the responsive cell lines, as detected by QRT-PCR analysis (Fig. 2). These data suggest that IL-27 may be exploited to restore HLA class I expression in SCLC cells without inducing a strong PD-L1-mediated adaptive immune resistance, which is a hallmark of IFN-γ [15].

### IL-27 signals through the STAT1 and STAT3 pathways in SCLC cells

**Fig. 3:**
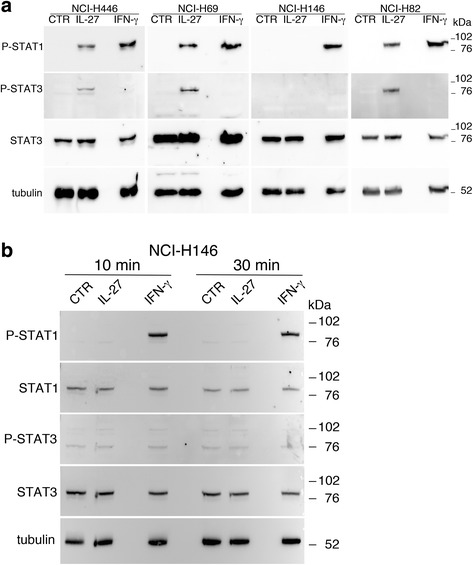
IL-27 mediates STAT1 and STAT3 phosphorylation in responsive SCLC cell lines. **a** Western blot analysis of tyrosine phosphorylated (P)-STAT1, P-STAT3 and total STAT3 proteins in SCLC cells cultured for 20 min with medium (CTR), IL-27 or IFN-γ. Total STAT3 and α-tubulin served as loading controls. No phosphorylation is detected in the IL-27-unresponsive NCI-H146 cells. **b** Analysis of P-STAT1, STAT1, P-STAT3 and STAT3 proteins in the IL-27-resistant cell line NCI-H146 cultured with medium alone (CTR), IL-27 or IFN-γ for 10 or 30 min. STAT1 phosphorylation was detected only following IFN-γ treatment, while phosphorylation of STAT3 was never detectable. Data are representative of two independent experiments with consistent findings

**Fig. 4:**
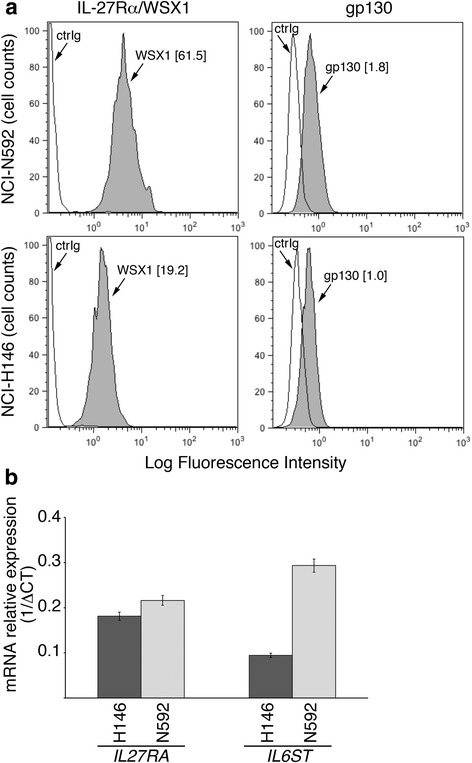
Expression of IL-27R subunits in IL-27-responsive (NCI-N592) and IL-27-non-responsive (NCI-H146) SCLC cell lines. **a** Cytofluorimetric analysis of surface IL-27Rα/WSX1 and GP130 receptor chain expression in IL-27-responsive (NCI-N592) and non-responsive (NCI-H146) SCLC cell lines. Isotype-matched Ig control is also shown (ctrIg). Numbers in brackets represent MFI values, calculated as above. **b** QRT-PCR analysis of IL-27Rα (*IL27RA*) and GP130 (*IL6ST*) receptor chain mRNA expression in IL-27-responsive (NCI-N592) and non-responsive (NCI-H146, dark histogram) SCLC cell lines. Data are expressed as 1/ΔCT relative to *POLR2A* housekeeping gene. Error bars represent SD in one representative experiment out of two with consistent data

Next, we analyzed IL-27-mediated STAT signaling in SCLC cells, in comparison with IFN-γ. As shown in Fig. 3a and Additional file 1: Fig. S1, IL-27 mediated both STAT1 and STAT3 tyrosine phosphorylation in the responsive NCI-H446, NCI-H69, NCI-N592 and NCI-H82 cell lines. Conversely, no STAT1 and STAT3 phosphorylated forms were induced in the IL-27-unresponsive NCI-H146 cells. The lack of IL-27 signaling via STAT1 and STAT3 in NCI-H146 cells was further confirmed by examining different time points of stimulation (Fig. 3b). Differently from IL-27, IFN-γ induced a strong tyrosine phosphorylation of STAT1 while STAT3 phosphorylation was undetectable in all the cell lines tested, including the NCI-H146 cells (Fig. 3 and Additional file 1: Fig. S1). To address the unresponsiveness of NCI-H146 cells to IL-27, we first analyzed the IL-27R complex surface expression by immunofluorescence and flow-cytometry. As shown in Fig. 4a, NCI-H146 cells expressed about 3-fold less IL-27Rα/WSX1 chain than the IL-27-responsive NCI-N592 cells, based on Median-Fluorescence Intensity (MFI) values. The expression of the GP130 chain was also lower on the NCI-H146 cell surface than on NCI-N592. Accordingly, QRT-PCR analyses showed lower levels of *IL27RA* and *IL6ST* (GP130) mRNA in NCI-H146 cells (Fig. 4b).

### Probing GP130 receptor signaling by sIL-6R/IL-6 in IL-27-responsive and -unresponsive SCLC cell lines

**Fig. 5:**
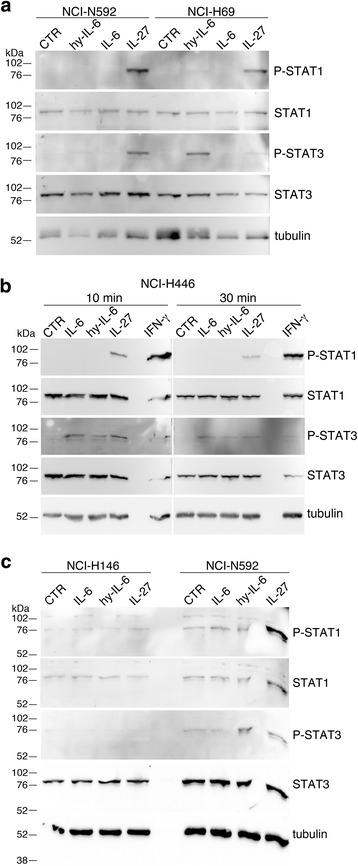
Comparative analysis of STAT1 and STAT3 phosphorylation in SCLC cell lines stimulated with different cytokines. **a** Western blot analysis of tyrosine phosphorylated (P)-STAT1 and P-STAT3 proteins in NCI-N592 and NCI-H69 SCLC cells cultured for 30 min with medium (CTR), sIL-6R/IL-6 (hy-IL-6), IL-6, or IL-27. Total STAT3 or STAT1 proteins and α-tubulin served as loading controls. **b** Kinetics (10 or 30 min) of STAT1 and STAT3 phosphorylation in the IL-27-sensitive cell line NCI-H446 cells cultured with medium alone (CTR), sIL-6R/IL-6 (hy-IL-6), IL-6, IL-27 or IFN-γ. **c)** Comparison of STAT1 and STAT3 phosphorylation in the IL-27-sensitive cell line NCI-N592 and in the IL-27-non-responsive NCI-H146 cells stimulated with medium alone (CTR), sIL-6R/IL-6 (hy-IL-6), IL-6, and IL-27

Since GP130 is a signaling receptor chain common to several cytokines of the IL-12 and IL-6 family, including IL-27 [30] and IL-6 [32], we tested the responsiveness of these cells to IL-6 or to sIL-6R/IL-6, a chimeric protein consisting of IL-6 linked to an extracellular portion of IL-6Rα, similar to “hyper-IL-6” [33]. Hyper-IL-6 mimics natural IL-6/IL-6Rα soluble complexes, which can directly activate GP130-expressing cells in the absence of surface IL-6Rα. Recombinant human IL-6 failed to activate STAT3 phosphorylation in NCI-N592, NCI-H69 and NCI-H146 cells, while it was capable of activating STAT1/3 signaling in the NCI-H446 cell line (Fig. 5). Indeed, IL-6Rα chain mRNA was less expressed in NCI-N592, NCI-H69 and NCI-H146 cells than in NCI-H446 by QRT-PCR analyses (Additional file 1: Fig. S2). Conversely, sIL-6R/IL-6 triggered STAT3 and to a much lesser extent STAT1 tyrosine-phosphorylation in NCI-N592, NCI-H69 and NCI-H446 cells (Fig. 5 and Additional file 1: Fig. S3). However, sIL-6R/IL-6 failed to activate STAT3 and STAT1 phosphorylation in NCI-H146 cells, further suggesting that, in these cells, unresponsiveness to IL-27 may be related to the very low expression of GP130 and/or its uncoupling with STAT signaling.

### sIL-6R/IL-6 fails to induce HLA class I expression and may interfere with IL-27 effects

**Fig. 6:**
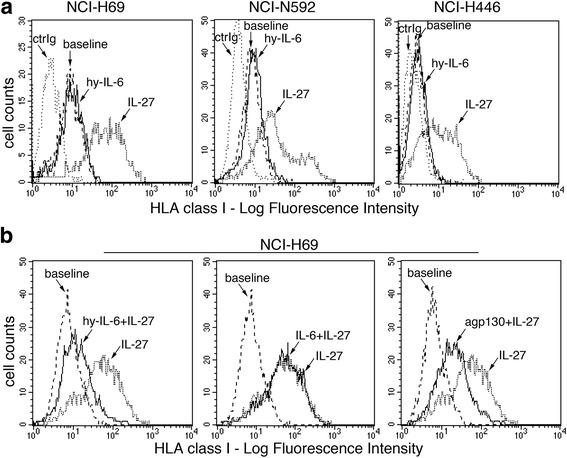
Immunofluorescence analysis of sIL-6R/IL-6 and IL-27 effects on HLA class I cell surface expression. **a** Cytofluorimetric analysis of membrane HLA class I expression in three SCLC cell lines, cultured in the presence of medium (baseline), IL-27 or sIL-6R/IL-6 (hy-IL-6), for 48 h. Isotype-matched Ig control is also shown (ctrIg). Different from IL-27, the GP130 engager sIL-6R/IL-6 does not up-regulate surface HLA class I expression in NCI-H69, NCI-N592 and NCI-H446 cell lines. **b** Analysis of HLA class I cell surface expression in NCI-H69 cells cultured for 48 h in the presence of medium (baseline), IL-27 (dotted line) or IL-27 plus sIL-6R/IL-6 (hy-IL-6), IL-6 or anti-GP130 mAb (agp130) (black line). sIL-6R/IL-6 and anti-GP130 negatively interfere with IL-27-mediated HLA class I antigen up-regulation, whereas IL-6 is ineffective

In view of the partially overlapping signaling properties of IL-27 and IL-6, we tested the possible effects of sIL-6R/IL-6 on HLA class I expression, in the IL-27- and sIL-6R/IL-6-responsive cells. As shown in Fig. 6, sIL-6R/IL-6 failed to induce HLA class I, which was up-regulated by IL-27 tested in parallel, in NCI-N592, NCI-H69 and NCI-H446 cells (Fig. 6a).

**Fig. 7:**
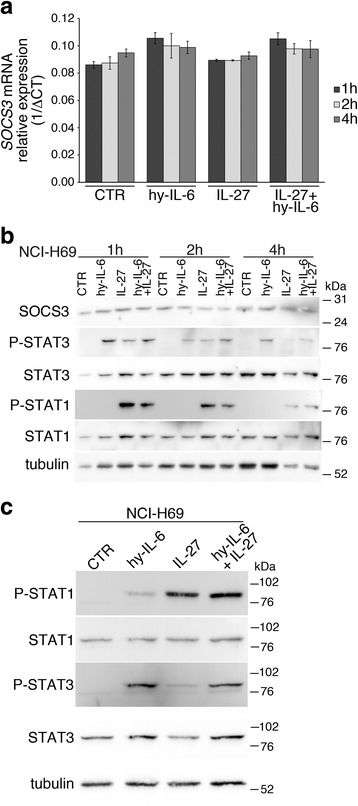
Analysis of SOCS3 expression and STAT1/3 phosphorylation following treatment with IL-27, sIL-6R/IL-6 or both cytokines. **a** sIL-6R/IL-6 (hy-IL-6) or IL-27, either alone or in combination, mediates only partial changes of *SOCS3* mRNA expression by QRT-PCR. Constitutive expression of mRNA is also evident. Data, normalized to *GAPDH* housekeeping gene, are expressed as 1/ΔCT. Error bars represent SD in one representative experiment out of two with consistent data. **b** Western blot analysis reveals constitutive SOCS3 expression (untreated control sample, CTR), which is marginally effected by sIL-6R/IL-6 (hy-IL-6) stimulation, at the indicated time intervals, in NCI-H69 cells. Tyrosine phosphorylated (P)-STAT1 and P-STAT3 proteins are analyzed as control of signal transduction. **c** Western blot analysis of NCI-H69 cells treated for 30 min with the indicated cytokines shows that IL-27 mediates stronger induction of P-STAT1, which is not inhibited by concomitant stimulation with sIL-6R/IL-6 (hy-IL-6). Tubulin served as loading control

Recent data indicate that IL-6 may interfere with IL-27 functions due to the induction of SOCS3 expression, which inhibits IL-27-mediated STAT signaling, in hepatocellular carcinoma cells [29]. Therefore, we analyzed whether IL-6 or sIL-6R/IL-6 may interfere with HLA class I induction by IL-27 in three different SCLC cell lines. Our data indicate that sIL-6R/IL-6 co-treatment strongly inhibits (about 90 ± 11%) IL-27-mediated HLA class I expression in NCI-H69 cells, while IL-6 was ineffective (Fig. 6b). Conversely only a marginal inhibition (10–20%) was observed in the other two cell lines tested (NCI-N592 and NCI-H446 cells), indicating heterogeneity in the sIL-6R/IL-6 inhibitory effect in different SCLC cells (data not shown). In addition, the use of an anti-GP130 neutralizing antibody had similar inhibitory effects on IL-27 activity, in NCI-H69 cells (Fig. 6b). However, pre-treatment of NCI-H69 cells followed by removal of sIL-6R/IL-6 was not effective (Additional file 1: Fig. S4). These data suggest that sIL-6R/IL-6 may attenuate IL-27 activity through a mechanism different from the induction of JAK/STAT signaling inhibitors, such as SOCS3, in the NCI-H69 model. Indeed, SOCS3 is constitutively expressed in NCI-H69 cells but its expression, as mRNA or protein, shows limited changes in response to sIL-6R/IL-6 stimulation either at short-term, i.e. after 1–4 h (Fig. 7a and b) or at longer times (Additional file 1: Fig. S5).

It is well known that several common effects of IFNs and IL-27 are mediated by STAT1 tyrosine phosphorylation, which is essential for the trans-activation of STAT1-sensitive genes. Although IL-27 also activates STAT3 phosphorylation, the STAT1-dependent, IFN-like effects prevail, in different cellular models [18, 29]. We then analyzed the effects of sIL-6R/IL-6 on IL-27-mediated STAT signaling. As shown in Fig. 7, sIL-6R/IL-6 is a stronger inducer of STAT3 than of STAT1 phosphorylation and, conversely, IL-27 mediates stronger STAT1 and weaker STAT3 phosphorylation, in NCI-H69 cells (Fig. 7c). The simultaneous stimulation with sIL-6R/IL-6 and IL-27 resulted in a strong phosphorylation of both STAT1 and STAT3, thus altering the phospho-STAT1/phospho-STAT3 balance.

## Discussion

In this study we show, for the first time, that cultured SCLC cells respond to IL-27 by activating STAT1, and to a lesser extent STAT3, tyrosine phosphorylation and by up-regulating HLA class I surface molecule expression. SCLC is an aggressive cancer, which usually shows down-regulated expression of HLA class I molecules and may therefore escape from immune recognition by CTLs [9, 10]. SCLC cells express tumor associated antigens, which can be recognized by CTLs including, for example, the cancer/testis antigens MAGE-1 and -3 [11], the ion channel gBK [12], recoverin [34] and the neuron-associated protein Hu [35]. Several lines of evidence suggest that CTLs may play a protective role in SCLC. Indeed, CTLs recognizing SCLC-associated antigens have been isolated from patients with long-term survival [36] eventually associated with concomitant autoimmunity due to antigen sharing by SCLC and normal tissues [34, 35]. In addition, the recent finding that SCLC patients respond to anti-PD-1 antibodies also supports a role for CTLs in SCLC immune control [5]. IFN-γ is able to restore HLA class I expression and SCLC antigen recognition by CTLs, in vitro, through the induction of different components of the antigen-presentation machinery, among which are TAP1 and TAP2 [11, 12]. However, IFN-γ is a stronger inducer of PD-L1 expression than IL-27, in SCLC and may therefore limit anti-tumor responses by PD-1 expressing CTLs in vivo. In this respect, IL-27 may offer a better chance to induce HLA class I expression with a limited effect on PD-L1 induction.

The induction of surface HLA class I expression by IL-27 and IFN-γ is related to the activation of the STAT1 signaling pathway, which is an essential mediator of both IFNs and IL-27 biological effects [19, 23, 26, 27]. Indeed, recent studies by proteomics [18] or gene expression profiling [29] in different cell types showed a broad overlap between IFN-γ and IL-27 effects, including the induction of several components of the HLA class I antigen presentation machinery. However, the NCI-H146 cell line was completely unresponsive to IL-27 both in terms of HLA class I induction and STAT1/3 signaling, suggesting heterogeneity in the response to IL-27 in different SCLC. In such cases, IFN-γ may be a more suitable agent to restore CTL responses against SCLC, as also the IL-27-resistant SCLC cells were sensitive to IFN-γ induction. Our present findings also suggested that IL-27 unresponsiveness could be related to specific alterations in the IL-27R complex. Indeed, the IL-27-resistant cell line NCI-H146 showed reduced expression of IL-27RA and GP130 receptor chains, and failed to activate STAT3 signaling in response to sIL-6R/IL-6, a direct agonist of GP130 [33].

IL-6 is a pro-inflammatory cytokine, which plays an important role also in the progression and immune-regulation in several types of cancers through the activation of the STAT3 pathway [37, 38]. Particularly, in SCLC patients IL-6 levels were elevated in the circulation and related to worse survival [31]. In addition, phosphorylated STAT3 was constitutively expressed in the SCLC tumors in vivo [39] and a very recent report supports a role for IL-6 produced by tumor-associated macrophages in the paracrine activation of STAT3, in SCLC [40]. Finally, NCI-H446 SCLC cells can produce IL-6 in response to Hypoxia-inducible factor 1α and express IL-6/STAT3-related genes [41]. Accordingly, in the present report, we could not detect constitutive tyrosine phosphorylation of STAT3 in all SCLC cell lines studied but IL-6 induced STAT3 activation only in the NCI-H446 cell line. The other SCLC cell lines failed to respond to IL-6 but three of them showed STAT3 activation upon sIL-6R/IL-6 stimulation. sIL-6R/IL-6 is similar to hyper-IL-6, an IL-6/IL-6Rα extracellular domain chimera, which mimics natural, soluble IL-6/IL-6Rα complexes and can directly activate the GP130 receptor in cells lacking IL-6Rα [33]. Indeed, IL-6 signaling through the GP130 chain requires the IL-6Rα, either as cell surface-bound molecule or in a soluble form in complex with IL-6 [42]. Accordingly, the IL-6-responsive cell line NCI-H446 expressed *IL6RA* gene, which was less expressed in the IL-6-unresponsive, sIL-6R/IL-6-sensitive NCI-N592, NCI-H69 and NCI-H146 cells. Our present data may prompt further studies to identify the potential role of natural IL-6/IL-6Rα complexes in the in vivo activation of STAT3 and in the pathogenesis of SCLC.

Previous findings indicated that GP130 signaling, induced by IL-6 ligands, may switch-off the responsiveness to IL-27, through the induction of the suppressor of cytokine signaling, SOCS3, in human hepatocellular carcinoma cells [29]. SOCS are P-STAT-inducible proteins, which act as potent blockers of STAT protein signaling in a feed back loop [43]. In view of the potential role of IL-6 ligands in SCLC, we tested whether sIL-6R/IL-6 may alter IL-27 effects in SCLC cells. The interference of sIL-6R/IL-6 was particularly evident in the NCI-H69 cell model, where the concomitant presence of sIL-6R/IL-6 inhibited IL-27-driven surface HLA class I expression by 90%. However, the inhibitory effect of IL-6 signaling seemed not related to SOCS3 induction in NCI-H69 cells because: i) pre-stimulation with sIL-6R/IL-6 followed by removal shortly before IL-27 stimulation failed to interfere with HLA class I induction, ii) SOCS3 is constitutively expressed by NCI-H69 cells and is not further induced by sIL-6R/IL-6 and iii) IL-27 induces STAT1 signaling also in the presence of sIL-6R/IL-6. The role of SOCS3 in cancer is still controversial, because SOCS3 has been reported as a tumor-suppressor or tumor-promoting molecule, in different tumors [44]. For example, in pancreatic cancer SOCS3 expression correlates with a better prognosis and overexpression of SOCS3 limits tumor growth, while SOCS3 silencing by promoter methylation has opposite effects [45]. On the other hand, constitutive SOCS3 expression has been reported in human melanomas, where it can inhibit responsiveness to IFNs and may contribute to resistance to IFN-treatment [46, 47]. Somehow surprisingly, this is not the case of NCI-H69 cells, which respond very well to both IFN-γ and IL-27, in spite of constitutive SOCS3 expression. An alternative explanation for the sIL-6R/IL-6-mediated inhibition of IL-27 effects, is that the concomitant signaling of sIL-6R/IL-6 and IL-27 alters the balance between tyrosine-phosphorylated forms of STAT1 and STAT3 (Fig. 7). Indeed, IL-27 is a weak inducer of STAT3 and a much better inducer of STAT1, whereas sIL-6R/IL-6 is a strong inducer of STAT3. During simultaneous stimulation by the two cytokines, phosphorylated STAT1/STAT3 heterodimer formation may be favored [48]. Although the functional role of such heterodimers is still poorly understood, their formation may reduce the availability of phospho-STAT1 homodimers, which are mediators of IL-27 intracellular functions. In the case of IFN-γ signaling, the inhibitory effect of sIL-6R/IL-6 is negligible, as IFN-γ is a stronger inducer of STAT1 phosphorylation than IL-27. Whatever the mechanism(s) of IL-6 interference with IL-27 biological activity, it may be overcome in therapeutic settings by the use of the IL-6/IL-6R blocking agents [49] or STAT3 inhibitors [50].

## Conclusions

In conclusion, our present data suggest that IL-27 might be exploited in immunotherapy approaches in advanced SCLC with down-regulated HLA expression. Previous clinical studies showed that IFN-γ therapy has no impact on survival in patients with SCLC in remission after standard therapies [51, 52] and this finding may relate to the ability of IFN-γ to induce immune-resistance through the PD-L1/PD-1 pathway. IL-27 might be combined with treatments aimed at restoring CTL responses, such as anti-PD-1/anti-PD-L1 therapy [5], and/or with agents blocking the IL-6/STAT3 pathway. In this respect IL-27 might be particularly useful at the onset of anti-PD-1 treatment to enhance CTL recognition of otherwise HLA class I-negative SCLC cells. At further anti-PD-1 administrations, up-regulation of HLA class I expression may rely on endogenous IFN-γ production by CTLs, once the immune response has been initiated.

## Additional file

Additional file 1: IL-27 mediates HLA class I up-regulation, which can be inhibited by the IL-6 pathway, in HLA-deficient Small Cell Lung Cancer cells. Supplementary Figures and Table. (DOCX 2856 kb)

## Abbreviations

CTL: Cytotoxic T Lymphocyte
GP130: Glycoprotein 130
HLA: Human Leukocyte Antigen
IFN-γ: Interferon-γ
IL-12: Interleukin-12
IL-18: Interleukin-18
IL-18BP: Interleukin-18 Binding Protein
IL-27: Interleukin-27
IL-27RA: Interleukin-27 Receptor A
IL-6: Interleukin-6
IL-6R: Interleukin-6 Receptor
NSCLC: Non-Small Cell Lung Cancer
PD-1: Programmed Death-1
PD-L1: Programmed Death-Ligand 1
SCLC: small cell lung cancer
sIL-6R/IL-6: IL-6Rα/IL-6 chimera
SOCS3: Suppressor of Cytokine Signaling 3
STAT1/3: Signal Transducer and Activator of Transcription 1/3
TAP1/2: Transporter associated with Antigen Processing 1/2
