# Supplementary material for: IL-27 mediates HLA class I up-regulation, which can be inhibited by the IL-6 pathway, in HLA-deficient Small Cell Lung Cancer cells
Source: J Exp Clin Cancer Res. 2017 Oct 11;36:140. doi: 10.1186/s13046-017-0608-z (PMC5637329; doi:10.1186/s13046-017-0608-z)
Supplement: Additional file 1: — IL-27 mediates HLA class I up-regulation, which can be inhibited by the IL-6 pathway, in HLA-deficient Small Cell Lung Cancer cells. Supplementary Figures and Table. (DOCX 2856 kb) [file 13046_2017_608_MOESM1_ESM.docx]

**G. Carbotti et al. Additional file 1**

**Table S1: QRT-PCR primers**

| **Gene** | **Forward primer** | **Reverse primer** |
| --- | --- | --- |
| *GAPDH* | GAAGGTGAAGGTCGGAGT | CATGGGTGGAATCATATTGGAA |
| *POLR2A* | GACAATGCAGAGAAGCTGG | GCAGGAAGACATCATCATCC |
| *TAP1* | GCAGTCAACTCCTGGACCACTA | CAAGGTTCCCACTGCTTACAGC |
| *TAP2* | ATGCCCTTCACAATAGCAGCGG | CCAAAACTGCGAACGGTCTGCA |
| *SOCS3* | CATCTCTGTCGGAAGACCGTCA | GCATCGTACTGGTCCAGGAACT |
| *IL6R* | GACTGTGCACTTGCTGGTGGAT | ACTTCCTCACCAAGAGCACAGC |
| *IL27RA (WSX1)* | CCGAGTTACACCTCCAGAGC | AGACATGGTGAGCTGTTCCC |
| *IL6ST (GP130)* | CACCCTGTATCACAGACTGGCA | TTCAGGGCTTCCTGGTCCATCA |

**Supplementary Figure 1**

**
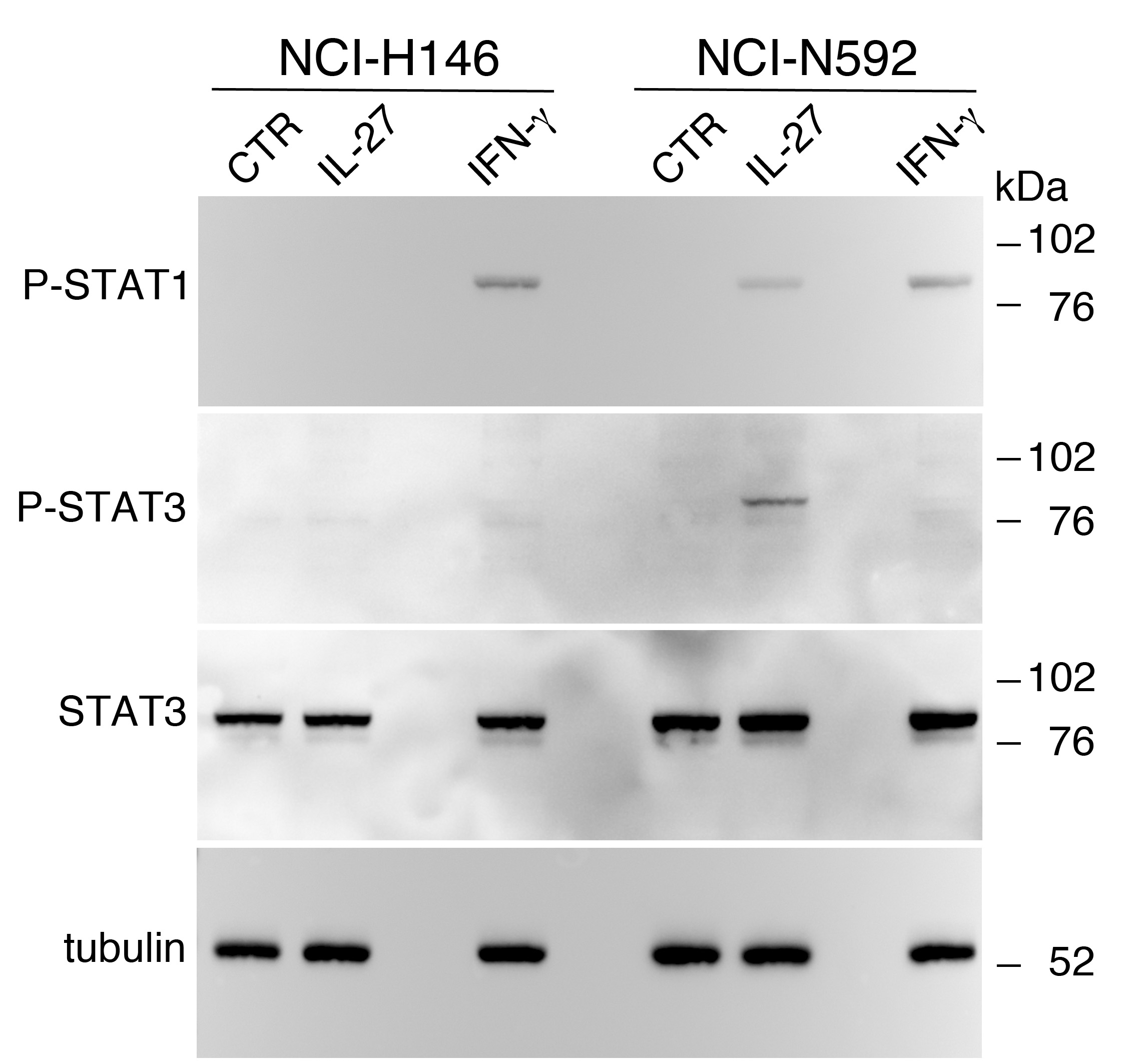
**

**Figure S1: IL-27 mediates STAT1 and STAT3 phosphorylation (P) in the responsive SCLC cell line NCI-N592 but not in the unresponsive NCI-H146 cells**

Western blot analysis of tyrosine phosphorylated (P)-STAT1, P-STAT3 and total STAT3 proteins in NCI-N592 and NCI-H146 SCLC cells cultured for 20 minutes with medium (CTR), IL-27 or IFN-γ. Total STAT3 and α-tubulin served as loading controls.

**Supplementary Figure 2**

**
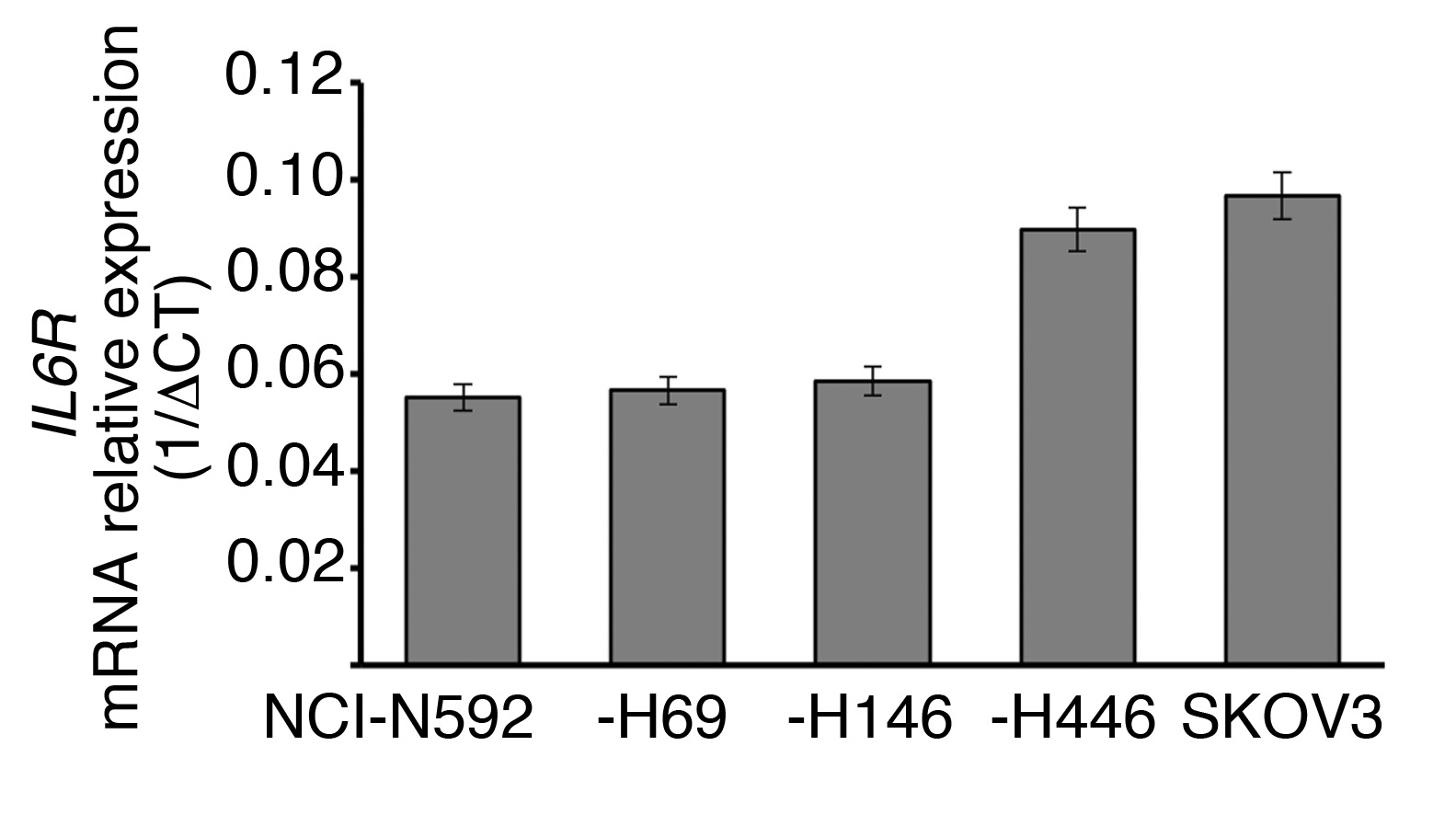
**

**Figure S2: Analysis of *IL6R* mRNA levels in SCLC cells**

QRT-PCR analysis of *IL6R* chain mRNA expression in IL-6-responsive (NCI-H446) and unresponsive (NCI-H146, -H69 and -N592) SCLC cell lines. An *IL6R* expressing ovarian carcinoma cell line (SKOV3) is also added as positive control. Data are expressed as 1/ΔCT relative to *POLR2A* housekeeping gene. Error bars represent SD in one representative experiment out of two with consistent data.

**Supplementary Figure 3**

**
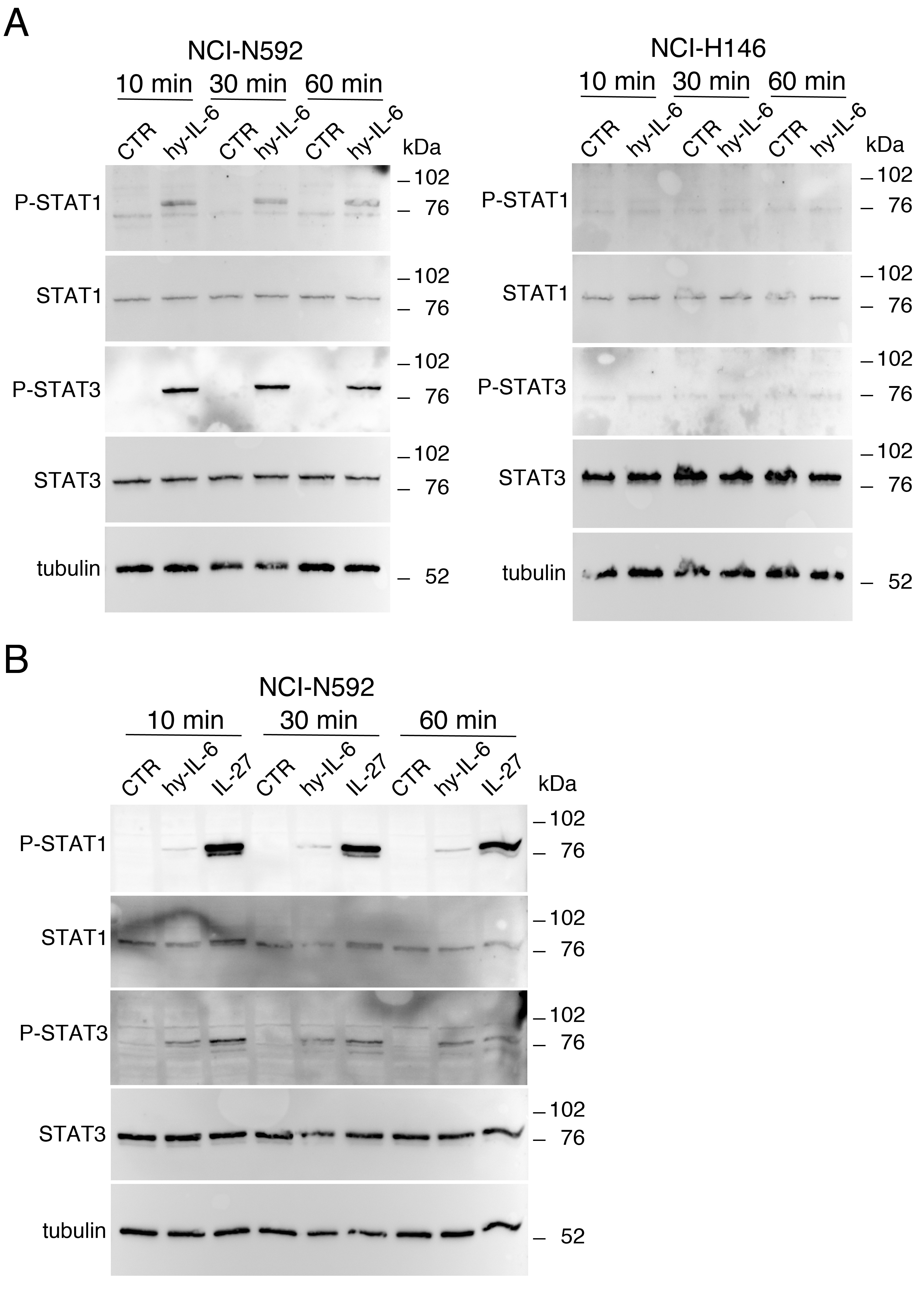
**

**Figure S3: Kinetics of STAT1 and STAT3 tyrosine phosphorylation induced by sIL-6R/IL-6**

The IL-27-sensitive cell line NCI-N592 are shown in comparison with the IL-27-resistant NCI-H146 cells **(A)** following stimulation with sIL-6R/IL-6 (hy-IL-6) for 10, 30 or 60 minutes. Weak STAT1 and stronger STAT3 phosphorylation (P) are observed only in the IL-27-responsive NCI-N592 cells. **B:** Comparative analysis of the kinetics of STAT1 and STAT3 phosphorylation upon stimulation with sIL-6R/IL-6 (hy-IL-6) and IL-27. It is evident that IL-27 is a stronger inducer of STAT1 phosphorylation than sIL-6R/IL-6.

**Supplementary Figure 4**

**
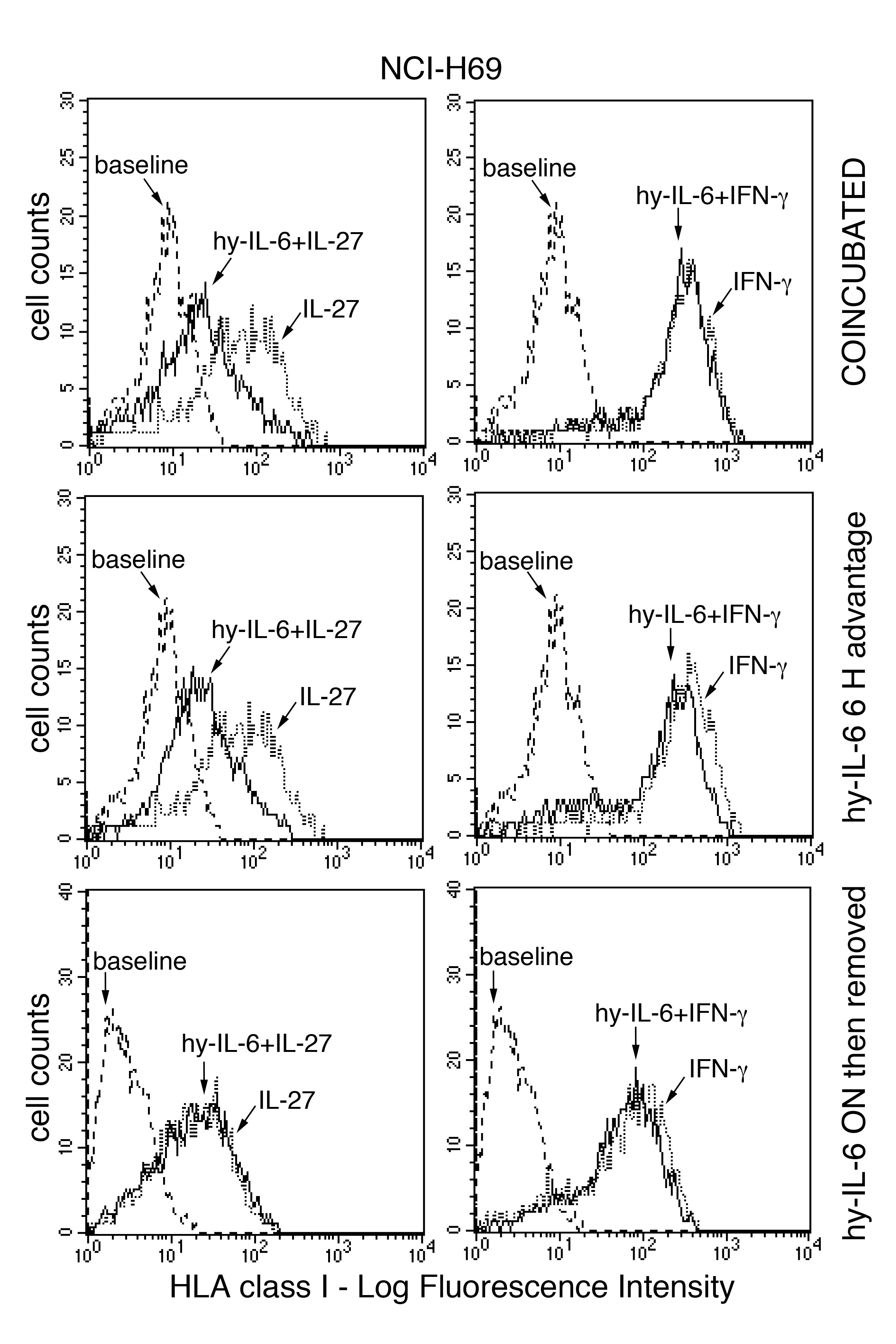
**

**Figure S4: Cytofluorimetric analysis of sIL-6R/IL-6** **and IL-27 effects on HLA class I surface expression in SCLC NCI-H69 cells**

SIL-6R/IL-6 (hy-IL-6) inhibits IL-27-mediated HLA class I antigen up-regulation, in NCI-H69 cells, when the two cytokines were simultaneously added (left upper panel) or when cells were pre-incubated with sIL-6R/IL-6 followed by the addition of IL-27 (left middle panel). On the opposite, overnight pre-treatment sIL-6R/IL-6 followed by its removal and subsequent addition of IL-27, resulted in no inhibitory effect (left lower panel). Induction of HLA class I by IFN-γ was only minimally influenced by any type of sIL-6R/IL-6 treatment (right panels).

**Supplementary Figure 5**

**
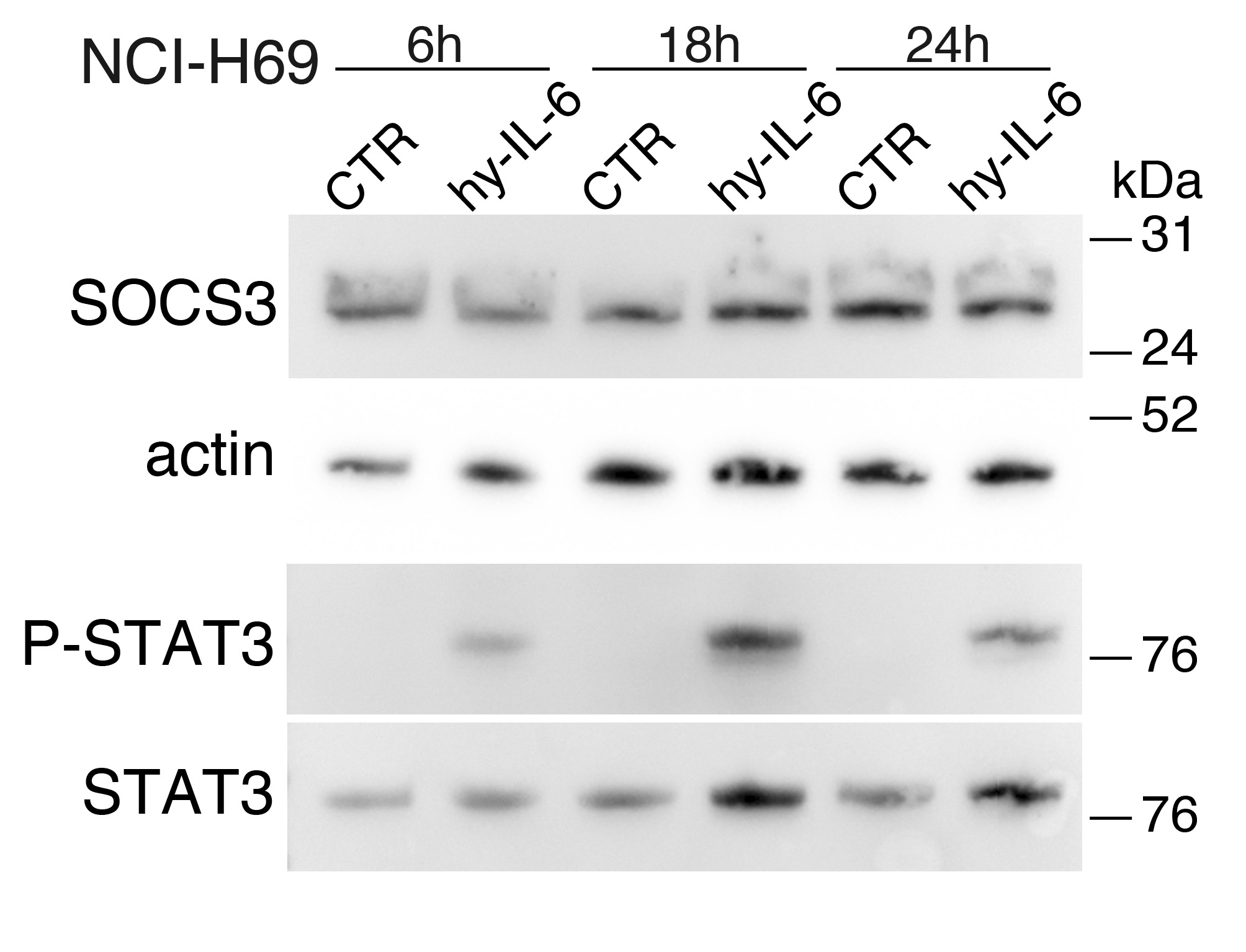
**

**Figure S5: Kinetics of SOCS3 levels and STAT3 tyrosine phosphorylation induced by sIL-6R/IL-6** **in NCI-H69 cells**

Western blot analysis of the kinetics of SOCS3 expression levels and STAT3 phosphorylation upon stimulation with sIL-6R/IL-6 (hy-IL-6) for 6, 18 and 24 hours in the NCI-H69 cell line. SOCS3 is constitutively expressed and only minor changes are observed in response to sIL-6R/IL-6 signaling, which is efficiently transduced, as shown by STAT3 phosphorylation.
